# Increased lower limb length ratio in patients with patellar instability

**DOI:** 10.1186/s13018-023-03720-w

**Published:** 2023-03-21

**Authors:** Maozheng Wei, Huijun Kang, Kuo Hao, Chongyi Fan, Shilun Li, Xingkai Wang, Fei Wang

**Affiliations:** Department of Orthopaedic Surgery, Third Hospital of Hebei Medical University, Shijiazhuang, 050051 Hebei China

**Keywords:** Patella alta, Lower limb length ratio, Patellar instability, Extensor moment arm

## Abstract

**Purpose:**

Patellar height is a risk factor for patellar instability, correlated with the tibia length/femur length (T/F) ratio. This study aimed to explore the changes in the T/F ratio in patients with patella instability and the potential correlation with the morphology of the patellofemoral joint and extensor moment arm.

**Method:**

A retrospective analysis was performed to assess the ratio of lower limb length morphological characteristics of the patellofemoral by full weight-bearing long-leg standing radiographs, magnetic resonance imaging, and computed tomography in 75 patients with patellar instability and 75 participants from a randomly selected control group from January 2020 to September 2021. A total of eight parts were measured, including mechanical tibia length/femur length (mT/F) ratio, anatomical tibia length/femur length (aT/F) ratio, hip–knee–ankle angle, femoral neck-shaft angle, femoral valgus cut angle, patellar height, Dejour classification, sulcus angle, trochlear angle, medial trochlear inclination, lateral trochlear inclination, patella tilt angle and patellar tendon moment arm to evaluate the difference of morphology between patient group and control groups.

**Results:**

The mT/F (0.840 ± 0.031 vs. 0.812 ± 0.026, *p* < 0.001) and aT/F (0.841 ± 0.033 vs. 0.808 ± 0.028, *p* < 0.001) ratios in the patient group were significantly greater than that in the control group. There was a significant correlation between patellar height and increased mT/F and aT/F ratios (*p* < 0.05).

**Conclusion:**

Patients with patellar instability had a larger lower limb length ratio, and the change in lower limb length ratio was correlated with patellar height.

*Level of evidence IV*.

## Introduction

Patellar instability is common and represents 3% of all knee injuries. It can result in significant limitations of activity and long-term arthritis [1, 2]. It occurred more frequently in the second decade of life, the overall risk of dislocation was seven in 100,000 annually, and it was elevated to 31 in 100,000 in the second decade of life [3, 4].

Specific anatomical abnormalities are closely associated with patellar instability; multiple anatomical malformations are combined deformities rather than being isolated. Trochlear dysplasia, patella alta, patellar tilt, and knee alignment play important roles in preventing lateral patella motion and keeping the patella congruent in the trochlear groove [3, 5, 6]. The simultaneous presence of patella alta can be found in 50–60% of cases of patellar instability, compared to approximately 3% in the normal population [7, 8]. The patella does not engage in the trochlea until higher knee flexion than normal, which leads to instability of the patellofemoral joint [9, 10]. Patella alta was often associated with a variety of anatomical abnormalities, including patella minor, patella tilt, and ligament laxity, thus, increasing the risk of subluxation or dislocation [11]. The causes and effects of patella alta are of great significance for the study of patellar instability.

The ratio of lower limb length is meant to be constant. Strecker et al. found a tibia-to-femur ratio of approximately 0.8 [12]. An increasing ratio of tibia/femur length was found to be significantly connected with ipsilateral hip and knee arthritis [13]. Dan et al. [14] found that adjusting the rabbit’s leg length ratio via epiphysiodesis of the distal femur and proximal tibia epiphysis resulted in developmental differences in patellar height, as well as morphological changes in the trochlea. The potential association between lower limb length ratio, patella alta, and the accompanying patellar instability is controversial and has been poorly reported.

This study aimed (1) to evaluate the difference in lower limb length ratio between the patellar instability and control groups; (2) to investigate the correlation between lower limb length ratio, patellar height, and patellofemoral joint morphology in patients with patellar instability; and (3) to explore the correlation between lower limb length ratio and knee extension moment arm. It was hypothesized that (1) lower limb length ratios were greater in patients with patellar instability, (2) the increased lower limb length ratio was collected with patella height and extensor moment arm.

## Method

We retrospectively analyzed patients with patellar instability from January 2020 to September 2021. The following were the inclusion criteria for the patellar instability group (1) recurrent patellar dislocation (≥ 3 times); (2) objective physical examination results, such as positive apprehension sign and positive J sign; and (3) imaging evidence of dislocation or subluxation confirmed using magnetic resonance imaging (MRI) and sunrise patella views). Exclusion criteria include: (1) BMI > 30 kg/m^2^; (2) chondromalacia patella or patellofemoral arthritis; and (3) a history of knee surgery or trauma [15]. Patients in the control group consulted the orthopedic surgeon for a complaint unrelated to the patellofemoral joint, such as a slight soft tissue injury. The control group was matched by age and sex. This study was approved by the institutional review board, and informed consent was obtained from all participants.

### Radiographic parameters

**Fig. 1:**
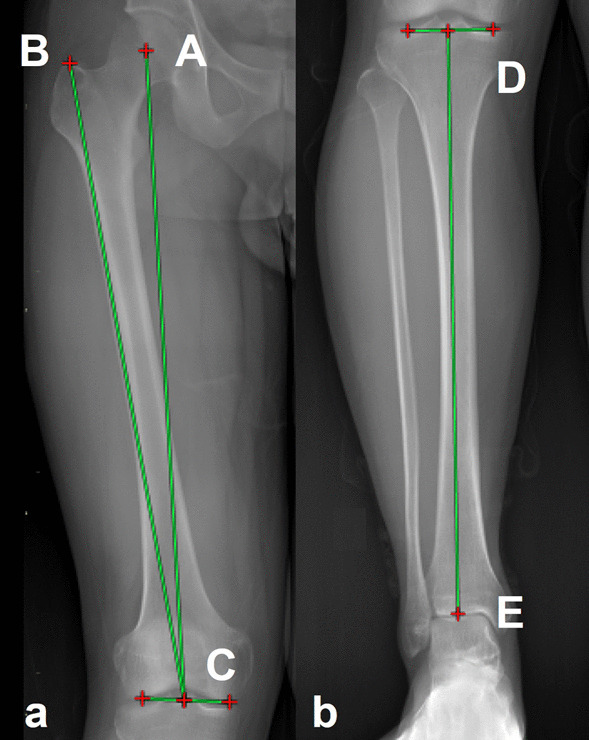
Measurement of lower limb length ratio. **a** The femoral length was measured from the superior center of the femoral head (A) to midpoint of the distal femoral condyle (C) when the mT/F ratio was calculated. The femoral length was measured from the top of the greater trochanter (B) to the center of the femoral condyles when the aT/F ratio was measured. **b** Tibia length was measured from the tibial plateau (D) to center of ankle joint (E)

**Fig. 2:**
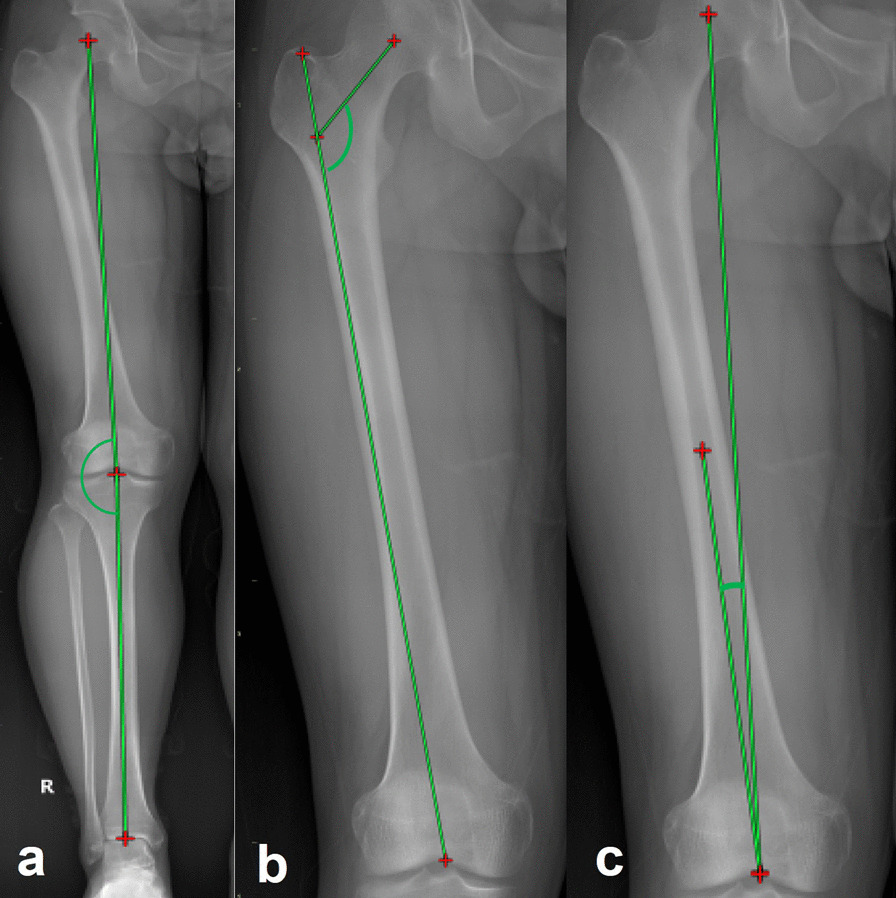
Measurement of coronal alignment. **a** HKA angle was defined as the angle between the line from the center of hip joint to the center of knee joint and the line from the center of knee joint to the center of ankle joint. **b** NSA measurement was generated by the intersection angle between the femoral neck and proximal femoral shaft axes. **c** The angulation between the femoral mechanical axis and the distal femoral anatomical axis was defined as VCA. *HKA* hip–knee–ankle angle, *NSA* femoral neck–shaft angle, *VCA* femoral valgus cut angle

**Fig. 3:**
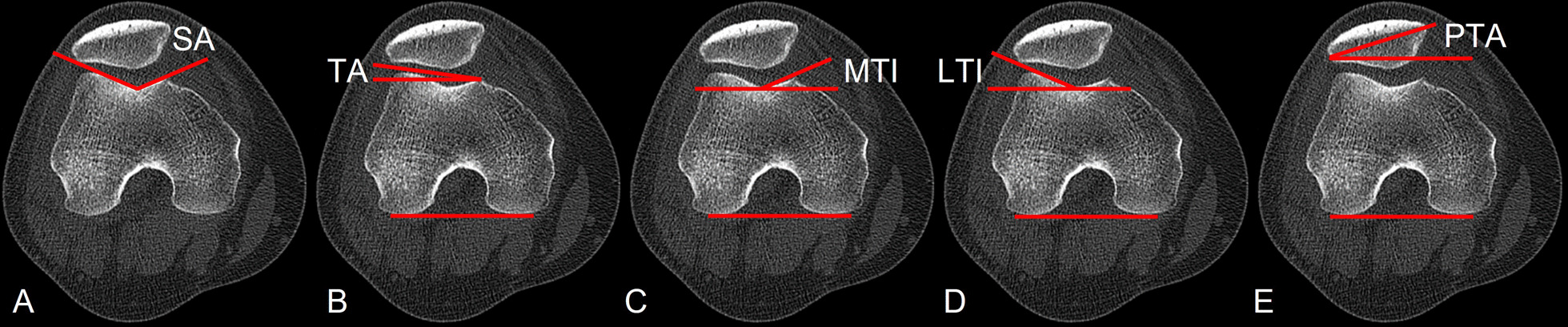
Schema of measured trochlea morphology and patellofemoral joint alignment. **A** Sulcus angle (SA) was the angle between the two lines alongside the lateral and medial trochlear surfaces from the lowest point of the sulcus. **B** Trochlear angle (TA) is the angle between the posterior condylar line (PCL) and anterior condylar line (ACL). The PCL is the line through the most posterior points of the posterior condyles, and the ACL is the line through the most anterior points of the anterior condyles. **C** The medial trochlear inclination (MTI) is the angle between the PCL and a line alongside the medial trochlear surface. **D** The lateral trochlear inclination (LTI) is the angle between the PCL and a line alongside the lateral trochlear surface. **E** A line called the maximal patella width line was drawn through the lateral and medial margins of the patella on the CT imagine in which the patella was widest. If the PCL and the maximal patella width line are not in the same slice, the maximal patella width line was duplicated to the former slice. PTA was the angle between the PCL and the maximal patella width line

All participants of the study underwent full weight-bearing long-leg standing and lateral knee radiographs according to a standardized protocol to avoid bias [16]. The femoral length was measured from the superior aspect of the femoral head to the center of the femoral condyles when the mechanical tibia length/femur length (mT/F) ratio was calculated. The femoral length was measured from the top of the greater trochanter to the center of the femoral condyles when the anatomic tibia length/femoral length (aT/F) ratio was measured. Tibia length was measured from the tibial plateau to the center of ankle joint (Fig. 1) [12, 13]. Hip–knee–ankle angle (HKA) was defined as the angle between the line from the center of hip joint to the center of knee joint and the line from the center of the knee joint to the center of the ankle joint. The angulation between the femoral mechanical axis and the distal femoral anatomical axis was defined as the distal femoral valgus cut angle (VCA) [17]. Neck-shaft angle (NSA) measurement was generated by the intersection angle between the femoral neck and proximal femoral shaft axes (Fig. 2) [18]. Four parameters indicated of the trochlea morphology, which included sulcus angle (SA), trochlear angle (TA), medial trochlear inclination (MTI) and lateral trochlear inclination (LTI), were measured on the axial CT images which had the largest posterior femoral condyles. SA was the angle between the two lines alongside the lateral and medial trochlear surfaces from the lowest point of the sulcus. TA is the angle between the posterior condylar line (PCL) and anterior condylar line (ACL). The PCL is the line through the most posterior points of the posterior condyles, and the ACL is the line through the most anterior points of the anterior condyles. The MTI is the angle between the PCL and a line alongside the medial trochlear surface, and the LTI is the angle between the PCL and a line alongside the lateral trochlear surface. The CT slice in which the patella was widest was selected, and a line called the maximal patella width line was drawn through the lateral and medial margins of the patella on this section. If the PCL and the maximal patella width line are not in the same slice, the maximal patella width line was duplicated to the PCL slice [19, 20]. Patella tilt angle (PTA) was the angle between the PCL and the maximal patella width line, and it was used to measure the rotation of the patella (Fig. 3) [21].

**Fig. 4:**
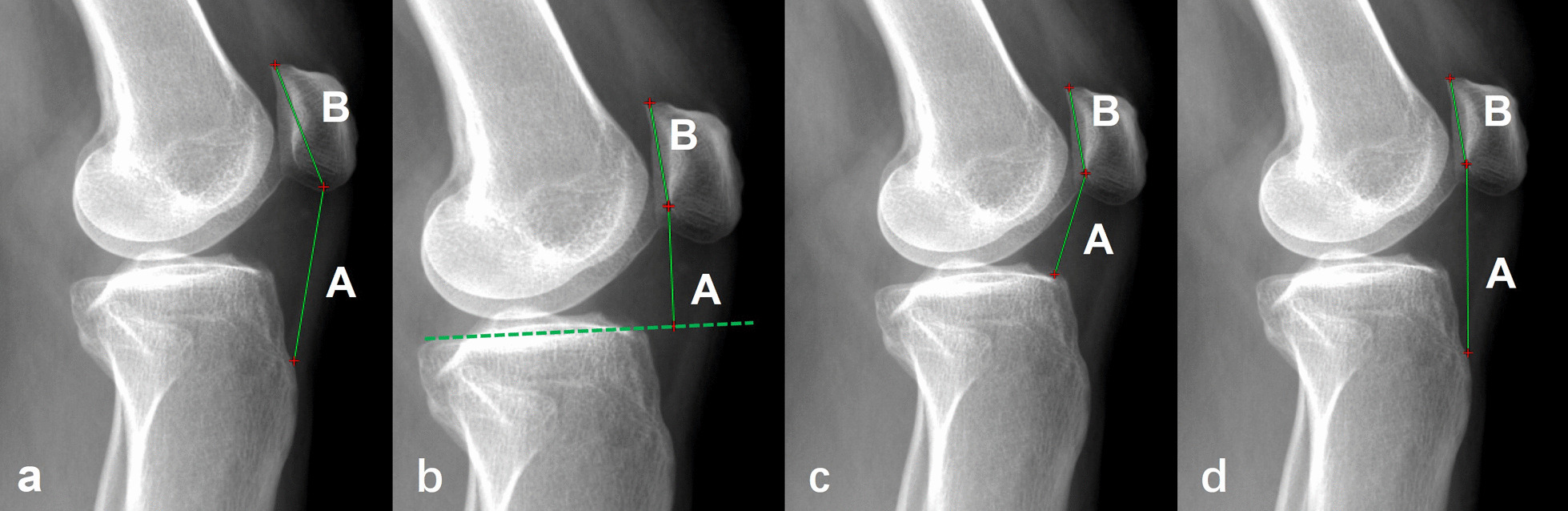
Patellar height measurement methods. **a** Insall–Salvati ratio: Ratio of the length of the patellar tendon (A) (measured from the distal pole of the patella to the tibial tuberosity) to the diagonal length of the patella (B) (measured from the distal pole to the proximal pole of the patella). **b** Blackburne–Peel ratio: Ratio of the height of the distal pole of the patellar articular surface above a tibial plateau line (A) to the articular surface length of the patella (B). **c** Caton–Deschamps ratio: the distance between the distal point of the patellar articular surface and the anterosuperior border of the tibia (A) divided by the length of the articular surface of the patella (B). **d** Modified Insall–Salvati ratio: Ratio of the distance between the distal end of the articular surface of the patella and the patellar tendon insertion on the tibia (A) to the length of the articular surface of the patella (B)

The four different methods for measuring patellar height were as follows: the Insall–Salvati (IS), Blackburne–Peel (BP), Caton–Deschamps (CD), and Modified Insall–Salvati (MIS) ratios (Fig. 4) [8].

### Computed tomography (CT) protocols

All patients underwent CT examination in the supine position, with 20° of knee flexion. The limbs were fixed using equipment to minimize motion. All examinations were performed using the same CT scanner (SOMATOM Sensation 16; Siemens Medical Solutions, Erlangen, Germany). The CT scanning parameters included a tube voltage of 120 kV, 100 effective mAs, 1-mm slice thickness, a gantry rotation time of 1 s, and a matrix size of 512. All measurements were performed using RadiAnt DICOM software (Medical, Poznan, Poland). Two experienced orthopedics assessed the Dejour classification of trochlear dysplasia that was assessed. Skeletal maturity was graded based on the distal femoral and proximal tibial epiphyseal, using one of the following categories: open, closing, or closed [22].

### MRI protocol

All study participants underwent full weight-bearing long-leg standing and lateral knee radiographs according to a standardized protocol to avoid bias. The MRI used for this study was obtained using a 1.5-T MRI (Sonata Magnetom, Siemens Medical Solutions, Erlangen, Germany) with the knee in or near full extension.

### Moment arm length measurements

**Fig. 5:**
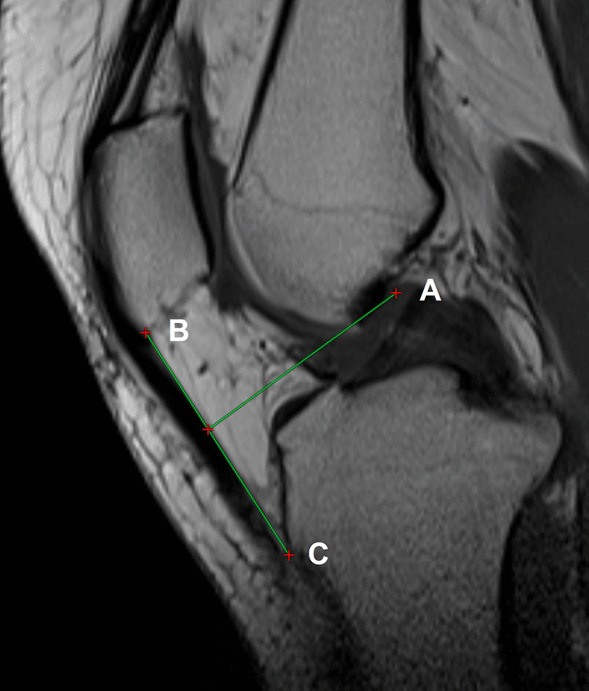
Measurement of PTMA. The PTMA is defined as the perpendicular distance from the intersection of the cruciate ligaments (A) to the patella ligament (B–C)

The patellar tendon moment arm (PTMA) was measured on MRI in the sagittal plane. It was defined as the perpendicular distance from the intersection of the cruciate ligaments to the patella ligament (Fig. 5) [23, 24].

### Statistical analysis

SPSS Statistics Package 21.0 (IBM, Armonk, New York, USA) was used for data analysis, and *p* < 0.05 was defined as statistically significant. To demonstrate alignment distributions for study and control groups, scatterplots for each population were prepared. The data were statistically analyzed by two researchers, and all the measured variables and data are described as mean ± standard deviation. The differences between the measurement data were analyzed using a paired-sample *t*-test, and Levene’s test was used to examine the data homogeneity. A linear regression test was performed to evaluate the correlation. Two independent investigators performed all the radiological measurements to reduce the observation bias. The inter- and intra-observer correlation coefficient (ICC) was calculated for all the radiological parameters. 0 indicated no agreement, 0–0.2 was a mild agreement, 0.2–0.4 was a fair agreement, 0.4–0.6 was a moderate agreement, 0.6–0.8 was a substantial agreement, and 0.8–1.0 was excellent agreement.

## Results

This study included 75 patients with patellar instability and 75 participants without patellofemoral joint disease as the control group. Table 1 shows the demographic data collected from all patients, including sex, age, and BMI. The skeletal maturity of all participants in the patellar instability and control groups was closed. Inter- and intra-observer ICC values were > 0.85 for all measurements (Table 2).

**Table 1:** Demographic characteristics of the participants

	Patients	Controls	*p* Value
Total participants, *n*	75	75	n.s
Age (years)	24.16 ± 7.302	24.60 ± 5.438	n.s
*Gender*			
Male	13	13	n.s
Female	62	62	
BMI (kg/m^2^)	24.35 ± 7.10	23.06 ± 6.71	n.s
*Side*			
Left	38	47	n.s
Right	37	28	

The data were shown as mean ± standard deviation

*BMI* body mass index, *n.s* not significant

**Table 2:** The inter-observer and intra-observer reliability of all measurements

Parameter	Inter-observer		Intra-observer	
	ICC	95%CI	ICC	95%CI
aT/F ratio	0.890	0.857–0.922	0.938	0.912–0.963
mT/F ratio	0.937	0.892–0.981	0.897	0.846–0.948
HKA	0.872	0.844–0.899	0.889	0.833–0.944
NSA	0.911	0.871–0.951	0.932	0.891–0.972
VCA	0.908	0.873–0.945	0.835	0.758–0.912
SA	0.947	0.927–0.967	0.971	0.951–0.991
TA	0.935	0.915–0.955	0.904	0.884–0.924
MTI	0.963	0.943–0.983	0.936	0.916–0.956
LTI	0.946	0.926–0.966	0.923	0.903–0.943
PTA	0.911	0.891–0.931	0.958	0.938–0.978
IS	0.930	0.881–0.978	0.878	0.827–0.928
BP	0.946	0.897–0.995	0.912	0.873–0.950
CD	0.903	0.865–0.947	0.869	0.816–0.922
MIS	0.896	0.862–0.934	0.930	0.889–0.970
PTMA	0.863	0.827–0.899	0.927	0.884–0.966

*aT/F* anatomical tibia length/femur length, *mT/F* mechanical tibia length/femur length, *HKA* hip–knee–ankle angle, *NSA* femoral neck-shaft angle, *VCA* femoral valgus cut angle, *SA* sulcus angle, *TA* trochlear angle, *MTI* medial trochlear inclination, *LTI* lateral trochlear inclination, *PTA* patella tilt angle, *IS* Insall–Salvati ratio, *BP* Blackburne–Peel ratio, *CD* Caton–Deschamps ratio, *MIS* modified Insall–Salvati ratio, *PTMA* patellar tendon moment arm

Patients with patellar instability had a significantly greater aT/F ratio than the control group (0.841 ± 0.033 vs. 0.808 ± 0.028, *p* < 0.001), and similar findings were also observed in the mT/F ratio (0.840 ± 0.031 vs. 0.812 ± 0.026, *p* < 0.001) (Table 3). The descriptive statistics of trochlea morphology and patellofemoral joint alignment is shown in Table 4. There was no significant difference between aT/F and mT/F ratios in both groups (patellar instability, *p* = 0.848, control, *p* = 0.468). HKA, NSA and VCA were not connected with the mT/F and aT/F ratios (Table 5).

**Table 3:** Lower limb length ratio of patients with patellar instability and control group

	Patients	Controls	*p* Value
aT/F ratio	0.841 ± 0.033	0.808 ± 0.028	*p* < 0.001
mT/F ratio	0.840 ± 0.031	0.812 ± 0.026	*p* < 0.001

The data were shown as mean ± standard deviation

*aT/F* anatomical tibia length/femur length, *mT/F* mechanical tibia length/femur length

**Table 4:** Descriptive statistics of trochlea morphology and patellofemoral joint alignment

	Patients	Controls	*p* Value
*Trochlea morphology*			
SA	161.112 ± 10.199	140.69 ± 7.983	*p* < 0.001
TA	8.749 ± 3.309	5.284 ± 1.467	*p* < 0.001
MTI	12.281 ± 5.874	10.209 ± 5.78	*p* = 0.037
LTI	16.369 ± 7.161	21.983 ± 5.706	*p* < 0.001
*Patellofemoral joint alignment*			
PTA	31.291 ± 9.413	13.843 ± 5.773	*p* < 0.001

The data were shown as mean ± standard deviation

*SA* sulcus angle, *TA* trochlear angle, *MTI* medial trochlear inclination, *LTI* lateral trochlear inclination, *PTA* patella tilt angle

**Table 5:** The correlation of lower limb length ratio to HKA, NSA, VCA and patellar height

Parameter	aT/F ratio	mT/F ratio
*Coronal alignment*		
HKA	0.454	0.345
NSA	0.581	0.290
VCA	0.675	0.378
*Evaluation of trochlear*		
SA	0.403	0.219
TA	0.425	0.211
MTI	0.379	0.222
LTI	0.329	0.173
*Patella tilt*		
PTA	0.387	0.213
*Patellar height*		
IS	0.002 (0.355)	0.001 (0.373)
BP	< 0.001 (0.480)	< 0.001 (0.562)
CD	< 0.001 (0.419)	< 0.001 (0.461)
MIS	0.054 (0.224)	0.009 (0.299)
PTMA	0.062	0.128

Data in table are* p* value, and data in parentheses are correlation coefficient (r)

*HKA* hip–knee–ankle angle, *NSA* femoral neck-shaft angle, *VCA* femoral valgus cut angle, *SA* sulcus angle, *TA* trochlear angle, *MTI* medial trochlear inclination, *LTI* lateral trochlear inclination, *PTA* patella tilt angle, *IS* Insall–Salvati ratio, *BP* Blackburne–Peel ratio, *CD* Caton–Deschamps ratio, *MIS* modified Insall–Salvati ratio, *PTMA* patellar tendon moment arm

**Fig. 6:**
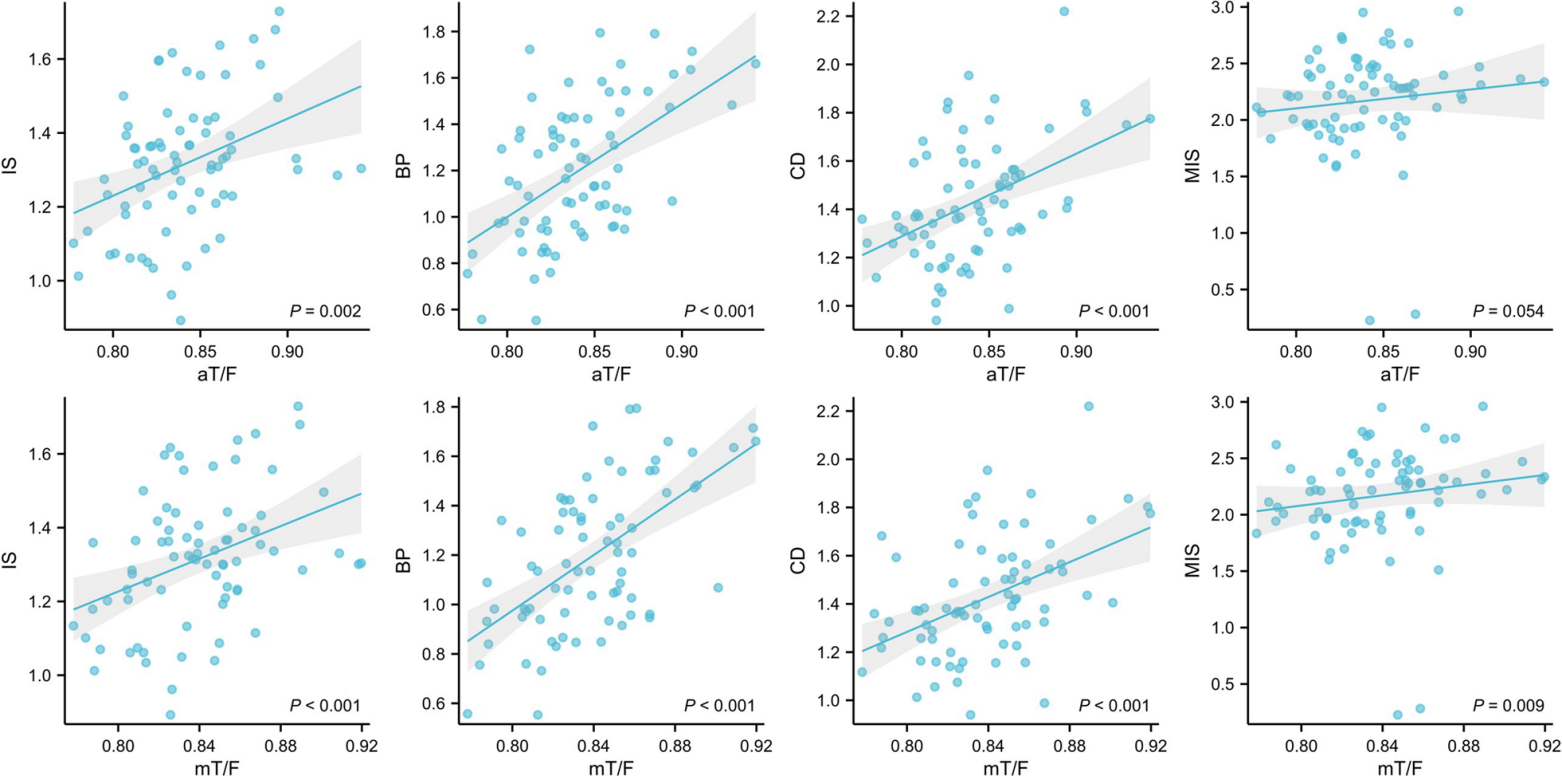
The correlation of lower limb length ratio to patellar height. *aT/F* anatomical tibia length/femur length, *mT/F* mechanical tibia length/femur, *IS* Insall–Salvati ratios, *BP* Blackburne–Peel ratios, *CD* Caton–Deschamps ratios and *MIS* Modified Insall–Salvati ratios

Patellar height is reported in Table 6. The mean patellar height was greater in the patellar instability group than that in the control group when applying the IS method (1.314 ± 0.180 vs. 0.973 ± 0.174, *p* < 0.001), BP method (1.197 ± 0.298 vs. 0.808 ± 0.373, *p* < 0.001), CD method (1.427 ± 0.248 vs. 0.902 ± 0.202, *p* < 0.001), MIS method (2.17 ± 0.446 vs. 1.347 ± 0.398, *p* < 0.001). Within IS, BP, and CD, the patellar height was collected with the anatomic limb length ratio. Within IS, BP, CD, and MIS, the patellar height was collected with the mechanical limb length ratio (Table 5) (Fig. 6).

**Table 6:** Results of patellar height in control group and patients with patellar instability

	Patients	Controls	*p* Value
IS	1.314 ± 0.180	0.973 ± 0.174	*p* < 0.001
BP	1.197 ± 0.298	0.808 ± 0.373	*p* < 0.001
CD	1.427 ± 0.248	0.902 ± 0.202	*p* < 0.001
MIS	2.170 ± 0.446	1.347 ± 0.398	*p* < 0.001

The data were shown as mean ± standard deviation

*IS* Insall–Salvati ratios, *BP* Blackburne–Peel ratios, *CD* Caton–Deschamps ratios, *MIS* modified Insall–Salvati ratios

The mean PTMA between patients with patellar instability and the control group was (4.41 ± 0.47 vs. 4.32 ± 0.45, *p* = 0.239), and no significant difference was observed between the two groups.

**Fig. 7:**
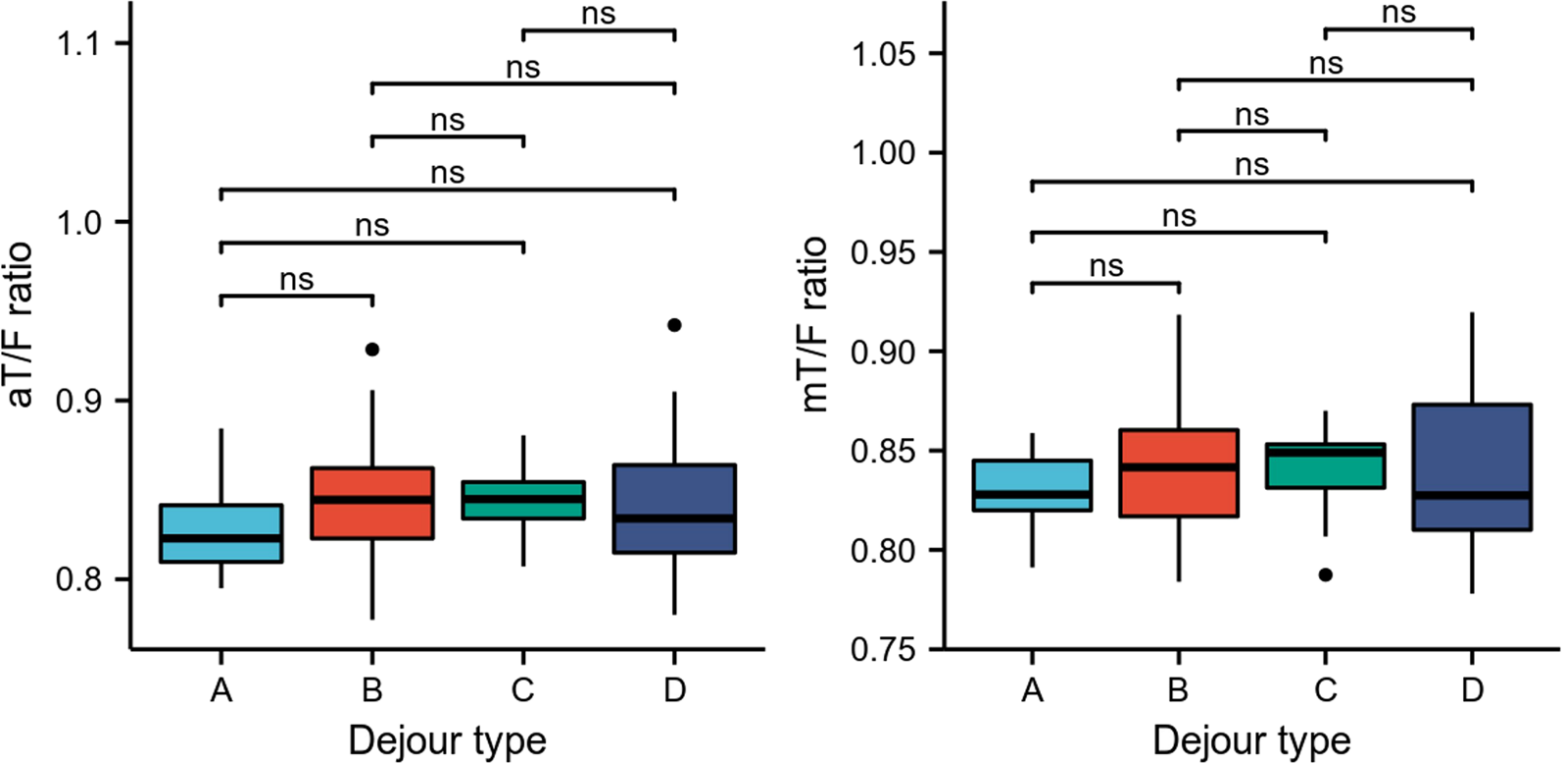
Distribution of aT/F ratio and mT/F ratio of different types of trochlear dysplasia. *aT/F* anatomical tibia length/femur length, *mT/F* mechanical tibia length/femur

**Fig. 8:**
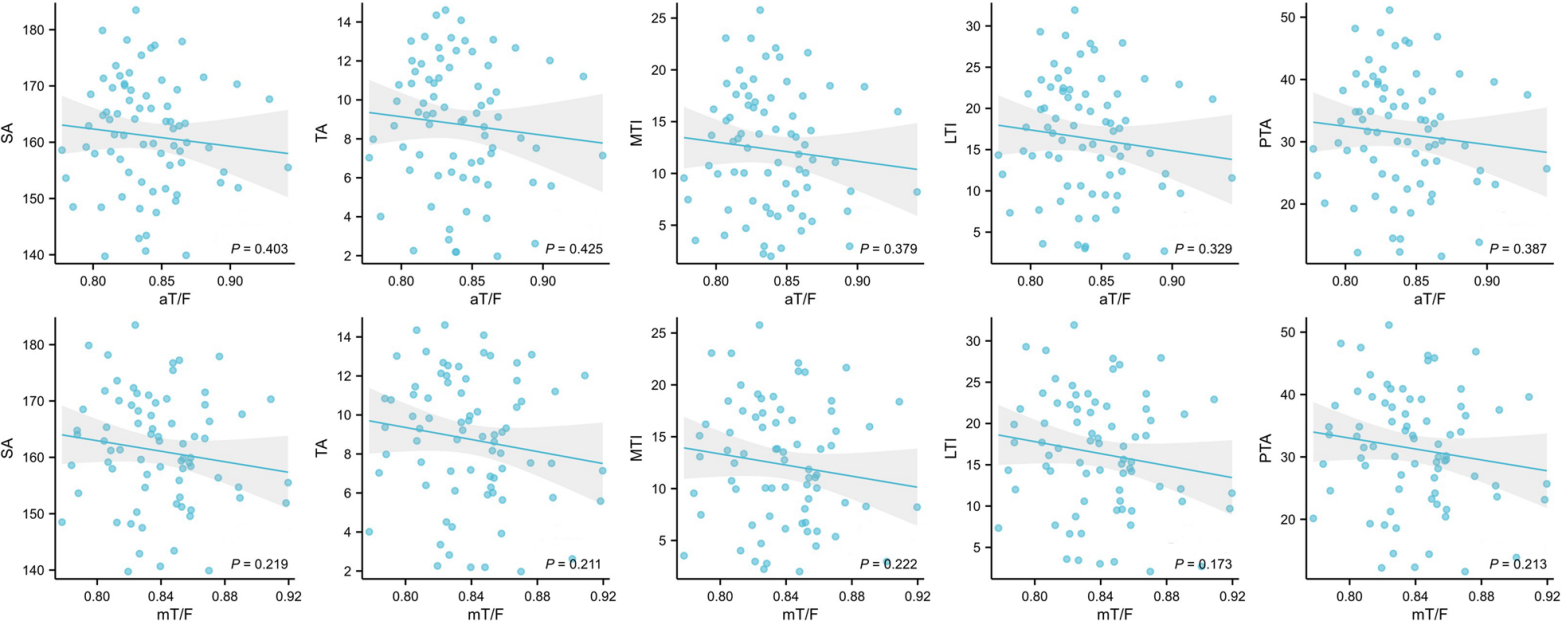
The correlation of lower limb length ratio to trochlea morphology and patellofemoral joint alignment

The patients with patellar instability were classified into Dejour type. The scoring of trochlear dysplasia resulted in the following distribution: type A n = 18, type B n = 22, type C n = 16, and type D n = 19. There was no significant difference in the aT/F ratio and mT/F ratio between Dejour grades (Fig. 7). There was no significant relationship between PTMA, sulcus angle, trochlear angle, medial trochlear inclination, lateral trochlear inclination, patella tilt angle and limb length ratio (Table 5) (Fig. 8).

## Discussion

The most significant study findings were that patients with patellar instability had an increased ratio of lower limb length ratio than the control group. There was a significant correlation between increased limb length ratio and patellar height in patients with patellar instability. The orthopedic surgeon should be aware of its possible influence on imaging diagnosis and surgical operation.

The ratio of lower limb length was greater in patients with patellar instability than that in the control group. Previous studies demonstrated an association between lower limb length ratio and the occurrence of lower limb diseases. Douglas et al. measured the ratio of limb length of the limb from 1152 cadavers and found that the physiological tibia/femur limb length ratio was close to 0.8 [12]. An increasing ratio of tibia/femur length was found to be significantly connected with ipsilateral hip and knee arthritis [13]. Several morphological risk factors, including trochlear dysplasia, patellar tilt, patella alta, increased femoral anteversion, and valgus alignment, have been linked to the occurrence of patellar instability; multiple anatomical malformations are combined deformities rather than isolated deformities [3, 5, 6, 25]. Liu et al. found that there were different posterior condyles and anterior morphological anatomy in patients with trochlear dysplasia, trochlear dysplasia was associated with a shorter lateral posterior condyle of the femur [26]. Yang et al. [5] found that distal femoral morphological dysplasia was associated with an increased femoral torsion in patients with patellar instability by morphological analysis based on CT. Currently, the etiology of patellar instability is unknown, and there is debate about the relationship between patellar instability and a variety of anatomical abnormalities. Our research sheds new light on patellar instability by examining changes in the lower limb length ratio in patients with patellar instability.

The mT/F and aT/F ratios were used to calculate the ratio between the length of the tibia, femoral shaft length, and the ratio of the femoral mechanical axis to evaluate the anatomical morphology of the femur and its influence on the mechanical properties of the lower limbs. Given that the coronal geometry of the femur may affect the femoral shaft and mechanical axis length, the VCA and NSA were measured concurrently, and the correlation between them and the femoral length was calculated. There was no significant difference between the mT/F and aT/F ratios. In patients with patellar instability, higher mT/F and aT/F ratios indicated a longer shaft and mechanical femoral axis length. This length change was unrelated to VCA or NSA.

Patella alta was one of the risk factors for patellar instability. Once the patella enters the trochlea at 20°–30° of flexion, the bony structure of the trochlea provides stability to the patellofemoral joint. With the presence of patella alta, the patella required a higher level of flexion to enter the trochlea than normal, resulting in an increased risk of recurrent patellar dislocation [8–10]. In this study, there were more patients with patella alta in the patellar instability group than those in the control group, consistent with previous reports. The correlation between patella alta and increased ratio of lower limb length indicated that the changes in patellar height and the ratio of lower limb length were combined deformities rather than isolated ones. In a collection consisting of the ratio of lower limbs and patella height, Dan et al. [14] adjusted the length of the femur and tibia by a rabbit epiphysiodesis model and found that the change of limb length ratio led to the difference in the patella height. The length of the patellar ligament may be the link between the lower limb length ratio and patellar height. Gloria et al. discovered a significant positive correlation between lower limb length and patellar ligament length in cadavers, with no gender difference [27]. The causes and effects of patella alta must be determined through the change of lower limb length ratio, which deserves more attention from surgeons.

The ability of the quadriceps to extend the knee depended on the moment arm of the knee extensor mechanism, which was measured using the patellar tendon moment arm (PTMA) of the knee joint [28]. PTMA was measured as the perpendicular distance from the intersection of the cruciate ligaments to the patellar ligament in the sagittal plane of MRI images [23]. Postoperative extensor mechanics in patients with patellar instability may be affected by surgery. Edmonds et al. [24] compared extensor realignment and medial patellofemoral ligament reconstruction patients to a control group and discovered that surgical adjustments altered joint reaction forces and effective moment arms. The patellar tendon moment arm was affected by patellar height. Lenhart et al. [27] found moderate patella alta acted to reduce quadriceps and patellar tendon loads in crouch gait, owing to an enhancement of the patellar tendon moment arms with alta in a flexed knee. Ward et al. [23] examined the MRI images of 27 participants and discovered that patellar ligament/quadriceps tendon force ratios and quadriceps effective moment arms were significantly larger in participants with patella alta, as was a more efficient knee extensor mechanism. There was also a link between PTMA and limb length. Thomas et al. investigated children's anthropometric characteristics and the patellar tendon arm and discovered a significant and strong correlation between the patellar tendon arm and anthropometric indicators. The PTMA could be predicted from the equation PTMA =  − 0.25 + 0.083 * tibia length + 0.02 * leg length at 85° of flexion [12]. According to the equation, a longer tibia length (lower limb length ratio) was associated with a larger knee extension arm. However, contrary to our hypothesis, no difference in PTMA was observed between the patellar instability and control groups, and no correlation was observed between PTMA and limb length ratio. Some factors may affect the observation. First, the knee patellar tendon moment arm measurement based on MRI was determined in the extension state. The study of Huntington et al. [29] was conducted in the knee flexion state, and previous studies had shown that the angle of knee flexion could cause potential influence. Secondly, there was a correlation between the PTMA and anatomical dimensions of the lower extremity. The measurement results could not be normalized based on the size of the femoral condyle in this study, owing to the instability of MRI for bone measurement.

In this study, trochlear dysplasia of varying severity was observed in patients with patellar instability. There was a close correlation between patellar instability and trochlear dysplasia, which was first proposed by Brattstroem in 1964 [28, 30]. Trochlear dysplasia was reported in approximately 85% of patients with recurrent patellar instability [29–33]. Wang et al. [34] found that subluxation or dislocation of the patella caused femoral trochlea dysplasia, and early reduction could prevent trochlear dysplasia in growing rabbits. The epiphysis is important in the development of the trochlea and changes in limb length; the peak incidence of patellar dislocation coincided with the closure time of the distal femoral and proximal tibial epiphyses [14, 25, 35]. Parikh et al. [36] retrospectively analyzed MRI of adolescent patients with trochlear dysplasia and found that the dysplastic trochlear was closely associated with the distal femoral epiphysis. The transverse epiphysis of the distal femur (the primary epiphysis) was responsible for longitudinal bone growth, and adverse factors leading to epiphyseal changes might affect limb length [37, 38]. There was no difference in the limb length ratio between patients with mild and severe trochlear dysplasia. Because all of the participants had closed epiphysis, the relationship between epiphysis change and limb length ratio was not observed in this study, which necessitates further research with participants who do not have closed epiphysis.

A variety of methods have been shown to be effective in determining the length of the lower limb, including supine CT and full-length radiography. Previous study found that there was an overall clinical similarity in the measurement of lower limb length from supine CT scan and full weight-bearing long-leg standing and lateral knee radiographs [39]. Following the ALARA principles “as low as reasonably achievable,’’ clinicians have an obligation to keep radiation exposure as low as possible [40]. Therefore, in this study, the length of the lower limbs was measured in the weight-bearing double lower limbs spliced in the anteroposterior position.

There were still some limitations to this study. First, this study was a non-randomized retrospective study, which has inherent defects. Second, MRI images from multiple angles were lacking. The direction and length of the extension arm of the knee joint were different in different flexion angles, which had a potential impact on the results of the study. Third, larger sample sizes and more age groups are needed to accurately reflect changes in the lower limb length ratio.

## Conclusion

This study shed light on anatomical anomalies in patients suffering from patellar instability. The lower limb length ratio of patients with patellar instability was greater than that of normal individuals, and a correlation between the change in lower limb length ratio and patellar height exists. Further studies are required to confirm this phenomenon and to determine the mechanism.

## Data Availability

The datasets used or analyzed during the current study are available from the corresponding author on reasonable request.

## Abbreviations

mT/F: Mechanical tibia length/femur length
aT/F: Anatomical tibia length/femur length
HKA: Hip–knee–ankle angle
NSA: Femoral neck-shaft angle
VCA: Femoral valgus cut angle
PTMA: Patellar tendon moment arm
IS: Insall–Salvati ratios
BP: Blackburne–Peel ratios
CD: Caton–Deschamps ratios
MIS: Modified Insall–Salvati ratios
CT: Computed tomography
MRI: Magnetic resonance imaging
ICC: Interobserver correlation coefficient
BMI: Body mass index
SA: Sulcus angle
TA: Trochlear angle
MTI: Medial trochlear inclination
LTI: Lateral trochlear inclination
PTA: Patella tilt angle
